# Identification and credentialing of patient-derived xenograft models of invasive lobular carcinoma

**DOI:** 10.1242/dmm.052710

**Published:** 2026-04-29

**Authors:** Jagmohan Hooda, Jennifer M. Atkinson, Osama Shiraz Shah, Megan Yates, Daniel D. Brown, Morgan DeBerry, Stefano Cairo, Paolo Schiavini, Hsiu-Wen Tsai, Marianna Zipeto, Rohit Bhargava, Steffi Oesterreich, Adrian V. Lee

**Affiliations:** ^1^ Women's Cancer Research Center, University of Pittsburgh Medical Center (UPMC) Hillman Cancer Center (HCC), Magee-Womens Research Institute, Pittsburgh, PA 15213, USA; ^2^ Department of Pharmacology and Chemical Biology, University of Pittsburgh, Pittsburgh, PA 15213, USA; ^3^ Institute for Precision Medicine, University of Pittsburgh, Pittsburgh, PA 15213, USA; ^4^ Department of Pathology, University of Pittsburgh, Pittsburgh, PA 15213, USA; ^5^ Champions Oncology, Hackensack, NJ 20850, USA

**Keywords:** Invasive lobular breast cancer, Patient-derived xenograft, *CDH1*, Genomic profiling, Targeted therapy

## Abstract

Invasive lobular cancer (ILC) is the most common special breast cancer subtype, accounting for 10-15% of all cases. The pathognomonic feature of ILC is loss of E-cadherin (encoded by *CDH1*), leading to discohesive single-file growth. Although ILCs show better prognostic factors than ‘no special type’ (NST) breast cancer, patients with ILC have worse long-term outcomes. We identified and validated patient-derived xenograft (PDX) models of ILC from 122 breast cancer PDX models based on truncating *CDH1* mutations and/or low *CDH1* mRNA expression. Eight PDX models were selected for validation using immunohistochemistry for E-cadherin, p120, estrogen receptor, progesterone receptor and HER2. Seven models were confirmed as ILC, and one showed mixed NST-ILC features. Confirmed ILC PDX models showed enrichment of truncating *CDH1* mutations, significantly lower *CDH1* mRNA expression and predominantly luminal subtypes compared to NST models, consistent with human ILC characteristics. Commonly altered genes included *PIK3CA* (57%), *CDH1* (57%) and *TP53* (57%). These validated ILC PDX models provide valuable tools to advance understanding of ILC biology and support development of targeted therapies.

## Teaser

**Summary:** By mining an existing patient-derived xenograft bank with multi-omics and histopathology, we identify seven validated invasive lobular cancer models that fill a critical gap in lobular breast cancer research.

## INTRODUCTION

Breast cancer is a heterogeneous disease with numerous histological subtypes. The most common subtype is ‘no special type’ (NST), accounting for more than two-thirds of all breast cancers. Invasive lobular cancer (ILC) is the most common special subtype, accounting for 10-15% of all breast cancers (American Cancer Society Cancer Facts and Figures, 2025; Siegel et al., 2025). In the USA alone, there were an estimated 47,500 new cases of ILC in 2025, which represented ∼15% of all invasive breast cancer diagnoses (American Cancer Society Cancer Facts and Figures, 2025; Siegel et al., 2025). The pathognomonic feature of ILC is the loss of E-cadherin (encoded by the *CDH1* gene) and cytoplasmic p120 (encoded by *CTNND1*), which leads to a lack of adherens junctions and a unique single-file growth pattern of discohesive ILC cells (Berx et al., 1995; Christgen et al., 2019). This growth pattern reduces the likelihood of detection by mammography, resulting in late detection and larger tumors. Although ILCs show similar or even better prognostic factors than NST, patients with ILC have worse long-term outcomes (American Cancer Society Cancer Facts and Figures, 2025; Siegel et al., 2025; Christgen et al., 2019; Bozkurt et al., 2021; Oesterreich et al., 2022).

ILC has historically been understudied, in part due to the lack of appropriate research models (Oesterreich et al., 2022; Mouabbi et al., 2022). Because ILC affects ‘only’ 10-15% of all breast cancers, the majority of models have been generated from the more frequent subtype, NST or invasive ductal carcinoma (IDC) (Sflomos et al., 2021). For example, the Cancer Cell Line Encyclopedia (CCLE) contains 54 NST cell lines but only two ILC cell lines. Additionally, only a limited number of patient-derived xenograft (PDX) models are evident in the published literature. There is a critical need for additional *in vitro* and *in vivo* models to study ILC biology, as well as to test targeted therapies. ILC PDX models and patient-derived xenograft organoids (PDXOs) are particularly valuable tools to enable target validation and assess drug treatment response (Guillen et al., 2022).

The purpose of this study was to identify and characterize PDX models of ILC. Specifically, we used whole-exome sequencing (WES) and RNA-sequencing (RNAseq) data from 122 human breast cancer PDX models to identify putative ILC PDX models based upon the presence of truncating *CDH1* mutations and/or low *CDH1* mRNA expression. Next, we validated a subset of these potential models by immunohistochemistry (IHC) for E-cadherin, p120, estrogen receptor (ER; also known as ESR1), progesterone receptor (PR; also known as PGR) and HER2 (also known as ERBB2) and confirmed seven of these PDX models as ILC. The findings from this study provide evidence for new PDX models that may be utilized to provide insights into the molecular characteristics of human ILC and develop new treatment strategies.

## RESULTS

**Fig. 1.**
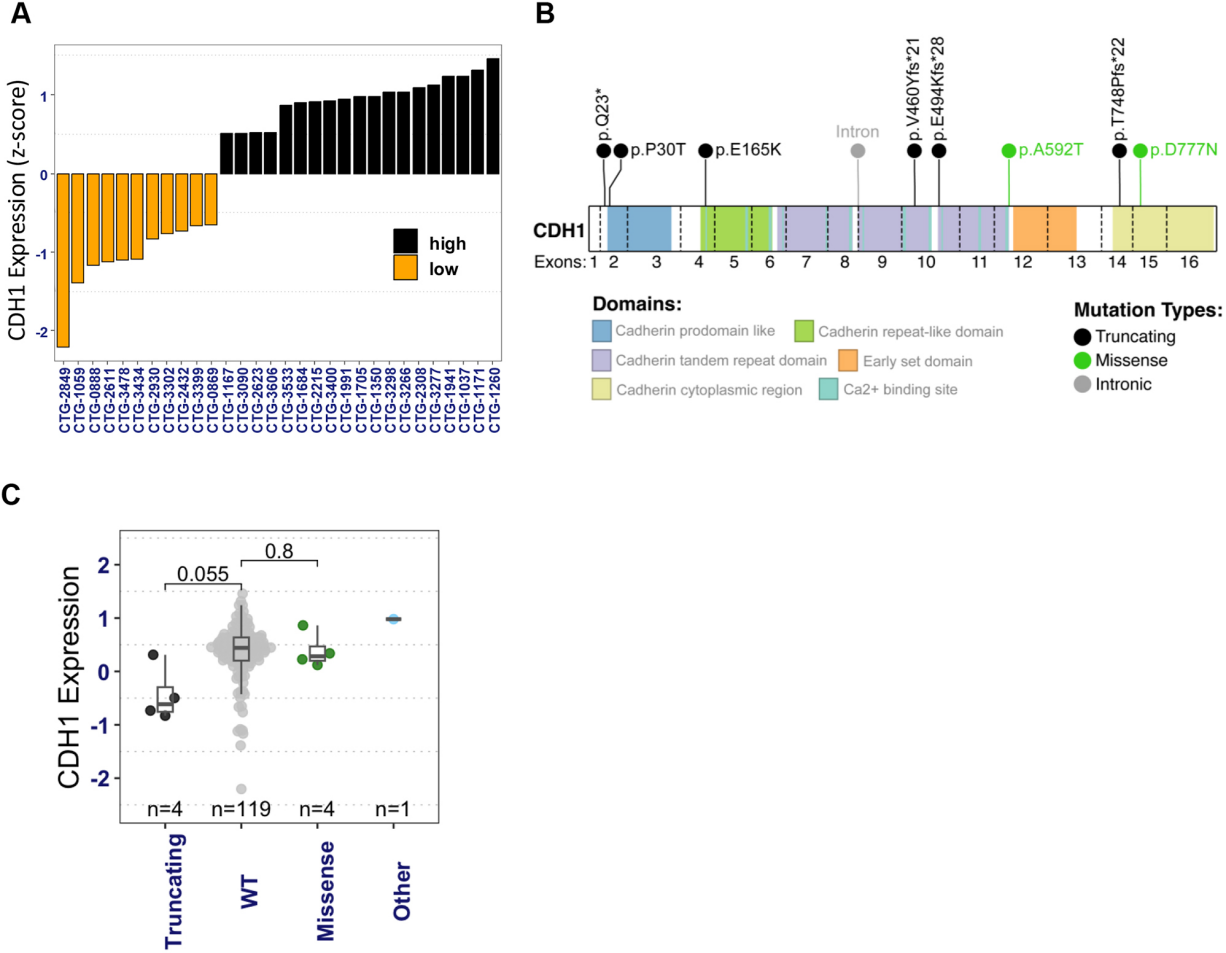
**Identification of potential invasive lobular cancer (ILC) patient-derived xenograft (PDX) models using multi-omic analysis of breast cancer PDX models.** (A) PDX models with highest and lowest *CDH1* mRNA levels (*z*-score). High *CDH1* expression models (black bars, *z*-score>0.5, *n*=19) and low *CDH1* expression models (orange bars, *z*-score≤−0.5, *n*=11) are shown. Low *CDH1* expression models (orange) were flagged as putative ILC. (B) Topography of *CDH1* mutations in PDX models: lollipop plot mapping somatic mutations to E-cadherin protein domains identified in champion PDX models. Exon boundaries are shown by dashed lines; numbers in each segment represent amino acid length. Lollipop height represents mutation frequency. Point colors denote mutation type (black, truncating; green, missense; gray, intronic). (C) *CDH1* expression in PDX models with *CDH1* mutations of various types [truncating, missense and other (intronic)] and or wild-type (WT) status. All PDX models with truncating *CDH1* mutations were flagged as putative ILC models. *P*-values were generated using two-sided Student's *t*-test.

We acquired WES and RNAseq data on 122 (seven ILC and 115 NST) breast cancer PDX models from Champions Oncology's Lumin portal. Principal component analysis of the RNAseq dataset, using the top 10% variably expressed genes, revealed two major clusters, as expected, representing the basal and luminal intrinsic molecular subtypes (Fig. S1A). We then identified potential ILC PDX models by examining their *CDH1* mRNA expression and *CDH1* gene mutations. Using RNAseq data, we identified 11 putative ILC PDX models with low *CDH1* mRNA levels (*z*-score≤−0.5) (Fig. 1A). Separately, using WES data, we identified nine *CDH1* variants across the PDX cohort: four truncating mutations in ILC models, two truncating mutations in NST models, two missense variants in NST models and one intronic variant, distributed across the *CDH1* gene body (Fig. 1B). The truncating mutations in ILC models (p.Q23*, p.V460Yfs21, p.T748Pfs22, p.E494Kfs28) are somatic variants with allele frequencies of 0.45-1.0, resulting in complete E-cadherin protein truncation. Three of these (p.Q23, p.T748Pfs22, p.E494Kfs28) have been previously reported in major breast cancer genomic cohorts (Molecular Taxonomy of Breast Cancer International Consortium, The Cancer Genome Atlas, Memorial Sloan Kettering), while p.V460Yfs*21 may represent a rare or novel variant. The missense variants p.A592T and p.D777N are germline variants classified as benign or likely to be benign in ClinVar. The somatic missense variant p.E165K (CTG-3533, NST) was not found in major databases searched and remains a variant of uncertain significance. Among the PDX models screened by combined criteria (*CDH1* mutations and/or low *CDH1* expression), seven models showed both *CDH1* alterations and low *CDH1* expression, while three models had low *CDH1* expression without detected mutations, and two models had mid-level *CDH1* expression with truncating *CDH1* alterations (Table S1). These models were flagged as putative ILC for histopathological validation. As expected, models with truncating *CDH1* mutations exhibited the lowest *CDH1* mRNA expression (Fig. 1C), a characteristic enriched in human ILC. In total, we identified 13 potential ILC PDX models using a combination of criteria including truncating *CDH1* mutations and/or low *CDH1* mRNA expression (Table S1).

Based on tissue availability and resources, we selected eight putative ILC PDX models for further histological and immunohistochemical analysis (Table S2). Notably, three of these putative ILC PDX models were annotated as being generated from human ILC, while the remaining five were classified as NST (i.e. ‘carcinoma’ or ‘ductal carcinoma’). Additionally, we selected two randomly chosen NST PDX models for comparison. Detailed clinical information – including tumor status (primary versus metastatic), harvest site and pre-biopsy treatment history for the samples used for ILC PDX model generation – is provided in Table S3. The majority of ILC PDX models (Table S3) were derived from metastatic lesions (9/13 putative models), with most source patients having received extensive prior therapies, including chemotherapy, endocrine therapy and/or targeted agents.

**Fig. 2.**
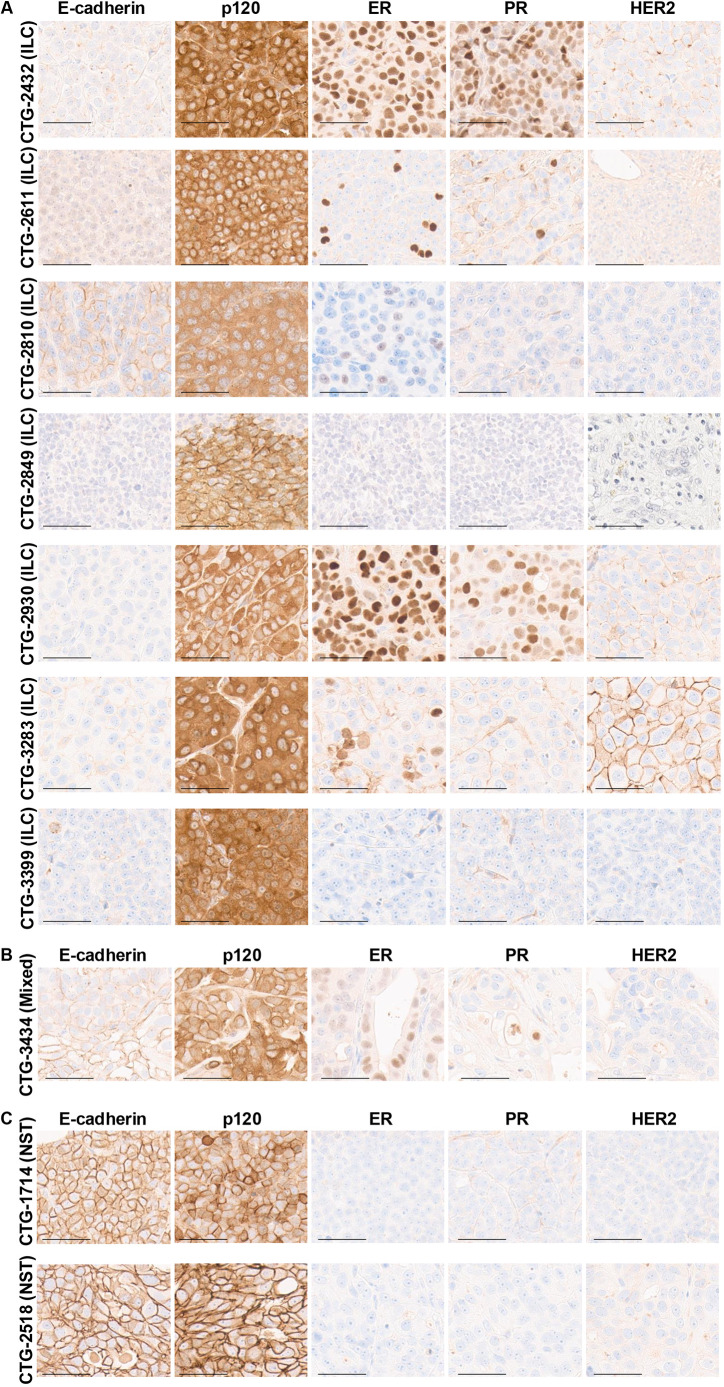
**Pathological assessment of PDX models identifying seven ILC PDX models in the Champions Oncology database.** The PDX formalin-fixed paraffin-embedded slides underwent staining with anti-E-cadherin, anti-p120, anti-ER, anti-PR or anti-HER2 antibodies on the Ventana Benchmark Ultra staining platform, and signal detection was conducted using Ultraview. A trained pathologist assessed the slides. (A) PDXs identified as ILC. (B) CTG-3434 was identified as a mixed ‘no special type’ (NST) ILC PDX. (C) NST PDXs used for comparison. Scale bars: 50 µm.

The ten PDX models were subjected to immunohistochemical analysis and pathology review for E-cadherin, p120, ER, PR and HER2 (see Table 1 and Fig. 2). IHC results showed that five of the candidate ILC models (CTG-2432, CTG-2849, CTG-2930, CTG-3283 and CTG-3399) exhibited a loss of E-cadherin expression and cytoplasmic localization of p120. Two further models (CTG-2611 and CTG-2810) showed aberrant membranous E-cadherin, but this was associated with cytoplasmic p120, indicating dysfunctional adherens junction. The anti-E-cadherin antibody Clone 36 used in this study, although extensively validated for ILC diagnosis (Dabbs et al., 2007), can show some cross-reactivity with P-cadherin, as recently documented (De Schepper et al., 2024). However, the critical diagnostic feature in these models is the combination of aberrant E-cadherin pattern with cytoplasmic p120 localization, which together with the molecular evidence (*CDH1* alterations and low *CDH1* expression) supports ILC classification. The remaining putative ILC model, CTG-3434, had an incomplete pattern of E-cadherin loss consistent with mixed NST ILC feature. Because of the mixed histology of CTG-3434, we attempted to grow this model in mice; however, the PDX failed to grow. In addition, Champions Oncology (personal communication) noted that this generation [passage (P)3] has a lower take rate. PDX fragments from an earlier passage had been cryopreserved in DMSO, and, upon thawing and re-engraftment, the later generation (P5) grew successfully. No growth factors were supplemented to support engraftment. Therefore, we analyzed tissue from the P5 generation, and, upon immunohistochemical analysis, the P5 generation was also identified as mixed NST ILC (Fig. 2B). As expected, the two NST control models (CTG-1714 and CTG-2518), exhibited membranous E-cadherin and p120 (Fig. 2C). Hematoxylin and Eosin histopathological evaluation revealed that most ILC PDX models displayed solid growth patterns with high-grade features, and only a minority showed classic lobular-like single-file architecture (Table S4). This predominance of solid growth patterns in PDX models, despite ILC molecular and immunohistochemical characteristics, likely reflects adaptation to the murine microenvironment and selection for aggressive, high-proliferative phenotypes during PDX establishment and passaging.

**Table 1.** Summary of histopathological analysis of the selected PDXs

PDX model	Tumor markers (PDX)	Selection criteria	E-cadherin IHC (PDX)	p120 IHC (PDX)	Histopathological evaluation
CTG-2432	ER^+^/PR^+^/HER2 2+ (HER2 non-amplified)	Truncating *CDH1* mutation (p.V460Yfs*21)	Negative	Cytoplasmic	Lobular
CTG-2611	ER^+^/PR^+^/HER2 1+	Low *CDH1* mRNA	Negative/aberrant (lobular pattern)	Cytoplasmic	Lobular
CTG-2810	ER^+^/PR^−^/HER2 1+	Truncating *CDH1* mutation (p.Q23*)	Weak membranous (lobular pattern)	Cytoplasmic	Lobular
CTG-2849	TNBC	Low *CDH1* mRNA	Negative	Cytoplasmic	Lobular
CTG-2930	ER^+^/PR^+^/HER2 1+	Truncating *CDH1* mutation (p.T748Pfs*22)	Negative	Cytoplasmic	Lobular
CTG-3283	ER^+^/PR^−^/HER2 2+ (HER2 non-amplified)	Truncating *CDH1* mutation (p.E494Kfs*28)	Negative	Cytoplasmic	Lobular
CTG-3399	TNBC	Low *CDH1* mRNA	Negative	Cytoplasmic	Lobular
CTG-1714	TNBC	NST comparison	Membranous	Membranous	Ductal
CTG-2518	TNBC	NST comparison	Membranous	Membranous	Ductal
CTG-3434	ER^+^/PR^−^ (<1%)/HER2 1+	Low *CDH1* mRNA	70% negative, 30% membranous	70% cytoplasmic, 30% membranous	Mixed NST ILC

IHC, immunohistochemistry; ILC, invasive lobular cancer; NST, ‘no special type’; PDX, patient-derived xenograft; TNBC, triple-negative breast cancer.

IHC for ER, PR and HER2 classified the ILC PDXs as ER^+^/HER2-low (*n*=5) and triple-negative breast cancer (TNBC) (*n*=2). Consistent with the HER2-low status, none of the models showed amplification of HER2 by fluorescence *in situ* hybridization, yet they all showed HER2 staining of 1+ or 2+. The prevalence of luminal B subtypes is consistent with our recent analysis of ILC cell lines as part of the invasive lobular cancer cell line encyclopedia (ICLE) project (Shah et al., 2023 preprint), in which we found the majority of cell lines to be luminal B and/or with elevated HER2 expression. It is likely that the growth *in vitro* or *in vivo* selects for cell lines and PDXs that have high HER2 and growth rate. In summary, out of the eight putative ILC PDX models, seven were confirmed as ILC, and one was characterized as mixed NST ILC PDX (CTG-3434). The immunohistochemical analysis provided additional evidence for the lobular histology of the PDX models and their classification as ER^+^/HER2-low (*n*=5) or TNBC (*n*=2).

**Fig. 3.**
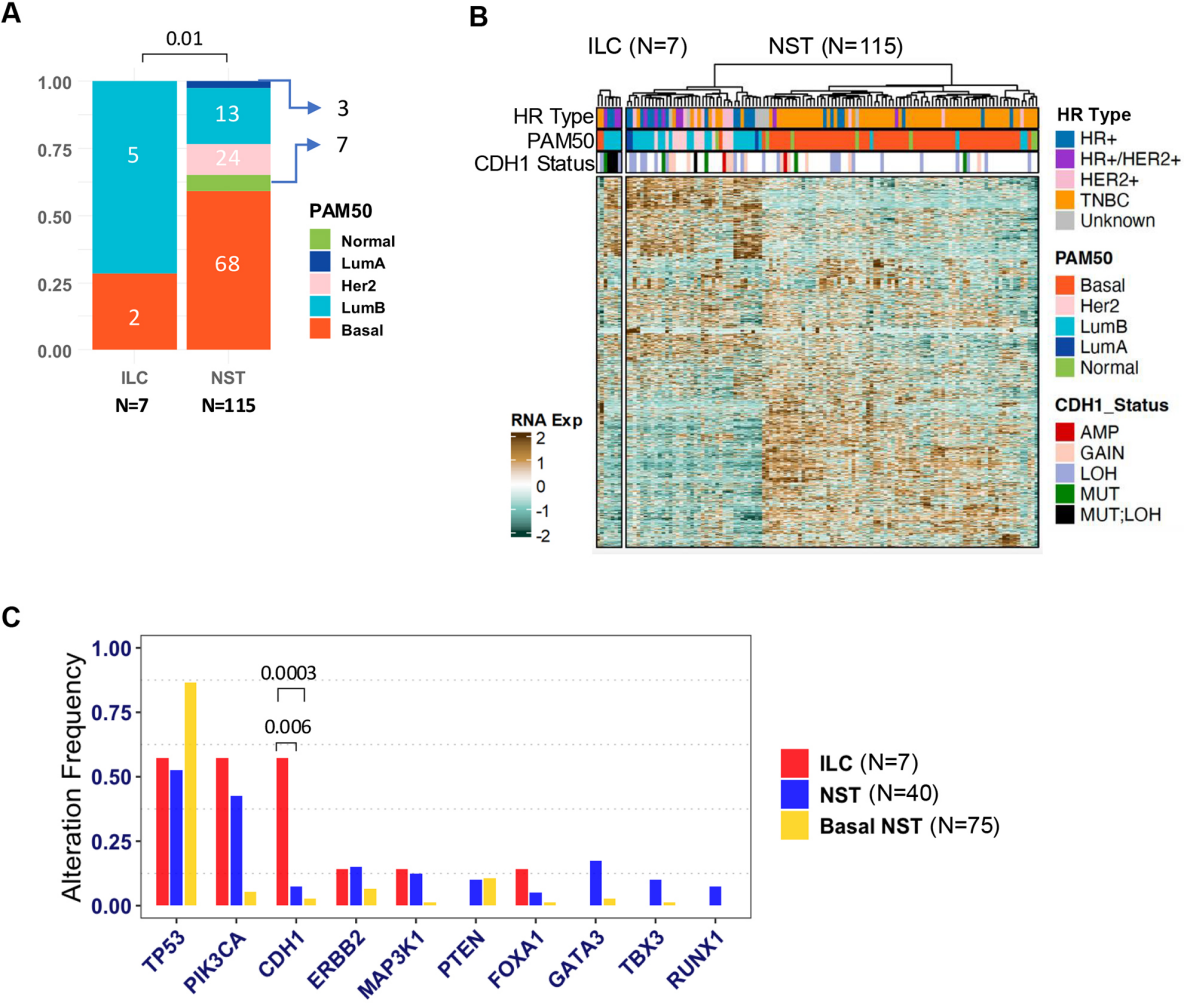
**Molecular features of ILC PDX models.** (A) PAM50 subtypes in ILC versus NST/IDC PDX models. Luminal subtypes were significantly enriched (Fisher's exact test, *P*=0.01) in ILC versus NST/IDC PDX models. (B) Gene expression heatmap of the top 15% variable genes for ILC (*n*=7) and NST (*n*=115) PDX models. Top annotation bars indicate hormone receptor (HR) type [HR^+^ (ER^+^ and/or PR^+^, HER2^−^), HR^+^/HER2^+^ (ER^+^ and/or PR^+^, HER2^+^), HER2^+^ (ER^−^/PR^−^, HER2^+^), triple-negative breast cancer (TNBC) or unknown (clinical status not available)], PAM50 molecular subtypes and *CDH1* alteration status. (C) Frequency of various gene alterations (MUT, MUT;LOH or MUT;GAIN) in all ILC, non-basal NST and basal NST PDX models across breast cancer genes frequently altered in breast patient tumors. *CDH1* alterations were significantly enriched in ILC versus non-basal NST (Fisher's exact test, *P*=0.006) and versus basal NST (Fisher's exact test, *P*=0.0003). Other gene alterations were not significantly different between ILC and non-basal NST PDX models. AMP, AMPLIFICATION; LOH, LOSS OF HETEROZYGOSITY; MUT, MUTANT.

We further analyzed the WES and RNAseq data to contrast the features between the ILC (*n*=7) and other NST (*n*=115) PDX models (Fig. 3). Most ILC models (5/7) were of luminal B intrinsic subtype, while the majority of NST models (68/115) were basal-like subtype (Fig. 3A; Fig. S1B). Notably, the luminal B subtype was significantly enriched in ILC versus NST PDX models (Fisher's exact test, *P*=0.005). Moreover, clustering of ILC and NST PDX models based upon the top 10% variable genes separated them into two clusters, i.e. basal and luminal/non-basal subtypes (Fig. 3B). Most ILC models showed a similar gene expression pattern to luminal/non-basal NST PDX models. In line with the selection criteria, ILC PDX models had lower levels of *CDH1* mRNA expression than NST PDX models (Fig. S1C). Similarly, differential enrichment analysis of alterations in key breast cancer genes (*TP53*, *CDH1*, *PIK3CA*, *ERBB2*, *FOXA1*, *MAPK31*, *TBX3*, *PTEN* and *GATA3*) between ILC and luminal/non-basal NST revealed significant (Fisher's exact test, *P*=0.005) enrichment of *CDH1* alterations in ILC models, as expected (Fig. 3C). Other top altered genes in ILC models included *TP53* (57%) and *PIK3CA* (57%), among others, while those in luminal/non-basal NST models included *TP53* (52%), *PIK3CA* (52%), *ERBB2* (42%) and *GATA3* (32%), among others (Table S5). Notably, ILC PDX models show a higher rate of alteration frequency in *TP53* gene than clinical ILC samples (∼8% in primary tumors and ∼20% metastatic tumors) (Shah et al., 2023 preprint). This is also true for ILC cell line models, which show 88% alteration frequency in *TP53* gene (Shah et al., 2023 preprint). The alteration frequency in *TP53* and *PIK3CA* genes was not significantly different between ILC and luminal/non-basal NST PDX models (Fig. 3C). As expected, the top altered gene in basal NST PDX models was *TP53* (87%) (Table S5) (Ciriello et al., 2015). In summary, ILC PDX models were predominantly of luminal subtype, showed enrichment for alterations in the *CDH1* gene and had significantly lower *CDH1* mRNA levels than NST PDX models.

## DISCUSSION

ILC is a unique subtype of breast cancer that accounts for 10-15% of all breast cancer but has historically been understudied, in part due to the lack of appropriate research models (Sflomos et al., 2021). To address this, additional *in vitro* and *in vivo* models are needed to study ILC biology and test targeted therapies. PDX models are critical *in vivo* models for breast cancer research (Sflomos et al., 2021; Kanaya et al., 2017). To address this need, we utilized WES and RNAseq datasets from Champions Oncology's Lumin portal to identify potential ILC PDX models and validated them using histopathological analyses. The study demonstrates how existing PDX banks can be interrogated to identify models of unique histological and molecular subtypes of breast cancer.

Our study identified seven PDX models of ILC based upon truncating *CDH1* mutation and/or low *CDH1* mRNA expression and confirmed using IHC and histopathological assessment. Findings from our study show that these ILC PDX models are predominantly of luminal intrinsic molecular subtype, show enrichment of *CDH1* mutations and exhibit lower levels of *CDH1* mRNA expression compared to NST, which is consistent with the characteristics of human ILC (McCart Reed et al., 2019). In addition to *CDH1* alterations, ILC PDX models also showed alterations in various other key breast cancer genes, including *TP53* and *PIK3CA*, among others, while lacking alterations in genes such as *ERBB2* and *GATA3*, which were more frequently altered in luminal/non-basal NST PDX models. A notable observation in our ILC PDX cohort is the elevated frequency of *TP53* mutations (57%) compared to primary human ILC tumors (∼7%) and even metastatic ILC (∼20%) (Shah et al., 2023 preprint). This enrichment likely reflects inherent selection biases in PDX establishment. PDXs are predominantly generated from aggressive, treatment-resistant tumors that have a higher probability of successful engraftment in immunocompromised mice. *TP53*-mutant tumors often exhibit more aggressive phenotypes, with enhanced proliferation and survival advantages, facilitating successful engraftment. This bias is not unique to our study; similar *TP53* enrichment has been reported in ILC cell line models (88% *TP53* alterations) (Shah et al., 2023 preprint), suggesting that *in vitro* and *in vivo* modeling systems both select for more aggressive, proliferative variants. Although this represents a limitation for studying classical, indolent ILC biology, these models are particularly valuable for investigating treatment-resistant and aggressive ILC variants, which represent significant clinical challenges. Our findings are consistent with previous studies describing molecular characteristics of ILC (Mouabbi et al., 2022; McCart Reed et al., 2019; Desmedt et al., 2016; Michaut et al., 2016).

The CTG-3434 PDX model was initially annotated as ILC and therefore included in our study; yet, we identified this model as mixed NST ILC upon histopathological analysis. Following challenges in expansion of this PDX line, P5 was successfully grown, and the tissue from P5 was characterized as mixed NST ILC, similar to P3 generation. The finding of mixed histology in PDX models is not unique and has been reported in other studies; for example, a study by DeRose et al. (2011) reported that some PDX models derived from breast cancer patients exhibited mixed histology, with both ductal and lobular components present in the same tumor (DeRose et al., 2011). In addition, the successful growth of the CTG-3434 PDX model in P5 is consistent with previous studies that have shown that PDX models can exhibit variable growth rates and success rates depending on the passage number (Pearson et al., 2016). Overall, the finding of mixed histology in the CTG-3434 PDX model highlights the complexity of developing ILC models and underscores the importance of careful histological analysis and comprehensive characterization of PDX models.

Our study has some limitations. First, the identified ILC PDX models were based on truncating *CDH1* mutation and/or low *CDH1* mRNA expression, which may not fully represent the heterogeneity of ILC, especially those cases that do not show either *CDH1* mutations or low *CDH1* mRNA levels but lack E-cadherin expression (Alexander et al., 2022). It is possible that using additional criteria such as mutations in other adherens junction genes (e.g. *CTNNA1*) or epigenetic changes may reveal further models. Second, the number of identified ILC PDX models is relatively small, primarily consisting of luminal B and basal-like subtypes owing to their growth advantage, rather than the more common luminal A subtype found in human ILCs. Further studies with larger sample sizes and more comprehensive characterization of ILC PDX models are needed to identify additional models for ILC and other special subtypes of breast cancer. Third, these PDX models were established by subcutaneous implantation in the flank rather than orthotopically in the mammary fat pad, which more faithfully recapitulates the native breast tumor stromal microenvironment (Sflomos et al., 2021). The absence of mammary-specific stromal cues may contribute to the predominance of solid growth patterns over classic single-file ILC architecture observed in these models, as detailed in the histopathological analysis (Table S4). Importantly, subcutaneous implantation has not been reported to alter the fundamental molecular identity of PDX tumors, and the ILC classification of these models is supported by their consistent molecular features, including truncating *CDH1* mutations, low *CDH1* mRNA expression and cytoplasmic p120 localization, irrespective of implantation site. Furthermore, the majority of these models were derived from metastatic lesions, where classical single-file architecture may already differ from that of primary ILC tumors.

Overall, these findings provide additional insights into the molecular characteristics of ILC compared to NST PDX models. Understanding of molecular subtypes, gene expression features and breast cancer gene mutations in the ILC PDX models could assist researchers in choosing the appropriate model for human ILC research and enable development of targeted therapies for this disease. Additionally, our study has implications for the development of new treatment strategies for ILC. The identification of seven ILC PDX models and one mixed NST ILC PDX model provides valuable tools for studying ILC biology and testing targeted therapies (Guillen et al., 2022; Kanaya et al., 2017). An important consideration for future work is that these ILC PDX models can serve as source material for generating PDXO models and, potentially, cell lines for *in vitro* studies. The development of cell lines from PDX models has proven more successful than direct derivation from primary patient tumors, as PDX-derived cells have already adapted to *ex vivo* growth conditions. Given the scarcity of ILC cell lines (only two in CCLE; Shah et al., 2023 preprint), establishing additional ILC cell lines and PDXOs from these characterized PDX models would provide valuable complementary *in vitro* tools for high-throughput drug screening and mechanistic studies. These characterized ILC PDX models will enable investigations beyond basic molecular confirmation. Specific research applications include testing ILC-targeted therapeutic strategies, particularly for pathways enriched in these models such as PI3K/AKT (*PIK3CA* mutations in 57% of models); investigating endocrine therapy resistance mechanisms in heavily pretreated metastatic disease; studying the biological significance of aberrant E-cadherin expression patterns in metastatic ILC; and examining the interplay between *TP53* mutations and ILC biology given the enriched *TP53* frequency in these models. Importantly, our study demonstrates how existing PDX banks with in-depth multi-omic and pathological analyses can be interrogated to identify models of unique histological and molecular subtypes of breast cancer.

### Conclusion

In conclusion, we characterized ten PDX models and identified seven ILC PDX models based upon *CDH1* mutation, low *CDH1* mRNA expression and histopathological analysis. Our findings highlight valuable new models for studying ILC biology and testing targeted therapies in PDX models. In addition, the genomic profiling techniques used in this study provide insights into the molecular characteristics of ILC models, which can help researchers utilize these models appropriately for their research. Further studies with larger sample sizes and more comprehensive characterization of ILC PDX models are needed to validate our findings and to develop further models for ILC research.

## MATERIALS AND METHODS

### PDX models

Champions Oncology is a preclinical research and clinical specialty testing provider that has developed over 1400 human PDX models (Tandon et al., 2023; Carter et al., 2023). Champions Oncology obtains consented patient samples to engraft, develop and characterize PDX models from a range of tumor types, primarily in immunocompromised mice. We queried the Champions Oncology collection of breast cancer models for potential PDX models of ILC. Detailed PDX establishment and propagation protocols have been previously described (Izumchenko et al., 2017). Briefly, models were established in athymic nude mice with tumor fragments implanted subcutaneously in the flank.

All molecular characterizations including WES, RNAseq and IHC were performed on early-passage PDX material (P2-4) to minimize molecular drift. These models are maintained and used for *in vivo* studies through P9. PDX tumor samples typically contain less than 10% mouse stromal content, ensuring that molecular profiling predominantly reflects human tumor cell characteristics.

PDX tumor growth rates vary by model, with tumors typically growing from implantation to 1000 mm³ in 30-100 days, reflecting the heterogeneity observed in the patient population. PDX engraftment success rates are influenced by tumor type and sample origin, with metastatic and recurrent disease showing higher take rates than primary tumors. Given the relative scarcity of ILC PDX models, precise take rate estimates for ILC specifically are difficult to determine. The PDX models are available for research use through Champions Oncology.

### Bioinformatics analysis

Clinical, genomic and transcriptomic data for breast cancer PDX models (*n*=128) were downloaded from Champions Oncology's Lumin portal (Tandon et al., 2023; Carter et al., 2023) on 22 February 2023, including clinical data, annotated mutation calls, copy number calls and RNA expression data. Six models were excluded from downstream analysis owing to incomplete clinical annotation, resulting in a final cohort of 122 PDX models (seven ILC, 115 NST) for all subsequent analyses. Detailed genomic and transcriptomic sequencing pipelines, including variant-calling methods, depth/allele-frequency thresholds, mouse DNA decontamination procedures and quality control metrics are publicly available at https://lumin.championsoncology.com/lumin/docs/NGSpipelines.html. Briefly, RNAseq reads were aligned to a combined human and mouse reference genome index (GRCh37 and GRCm38) using STAR aligner, followed by an *in silico* de-mousing step to remove mouse-derived reads prior to downstream analysis. De-mousing statistics, which serve as a proxy for tumor purity, are generated as part of the standard QC process for each model and are available upon request from Champions Oncology. All downstream analysis of these datasets was performed using R. Clinical, genomic and transcriptomic data were harmonized by model ID, and integrative data frames were generated. Silent mutations were excluded. Copy number calls were defined as GAIN (copy number, 1), LOSS OF HETEROZYGOSITY (LOH; copy number, −1), DIPLOID (copy number, 0), AMPLIFICATION (AMP; copy number, 2) and DELETION (DEL; copy number, −2). RNA expression data were log2-transformed transcripts per million (TPM) counts and further standardized to gene-wise *z*-scores. PAM50 (Prediction Analysis of Microarray 50) molecular subtypes were computed from RNA expression data using molecular subtyping function from the R package genefu (Gendoo et al., 2016). Mutation frequencies in key driver genes (*CDH1*, *PIK3CA*, *TP53*, *ERBB2*, *RUNX1*, *TBX3*, *PTEN*, *FOXA1*, *GATA3*, *MAP3K1*) were compared across ILC, NST and basal NST subsets.

Putative ILC models were defined based on the following criteria: presence of truncating *CDH1* mutations (frameshift, nonsense or splice site) and/or low *CDH1* mRNA (*z*-score<−0.5). Models clinically annotated as lobular disease were also included. A total of eight putative ILC PDX models were selected for further downstream histopathologic and immunohistochemical validation (see ‘Immunohistochemistry’ section).

All analyses are reproducible from the GitHub repository containing the R Markdown scripts and session information: https://github.com/leeoesterreich/2025_ILC_PDX_Multiomics.

### Statistical methods

Comparisons of gene expression distributions (e.g. *CDH1* expression between mutation types or histological subtypes) were performed using two-sided Student's *t*-tests (ggpubr::stat_compare_means), with significance annotated on figures. Fisher's exact tests were applied to assess enrichment of PAM50 luminal versus non-luminal subtypes in ILC versus NST, and to test for enrichment of driver gene mutations in ILC relative to NST and basal NST subgroups. Odds ratios and *P*-values from Fisher's exact tests are reported in the main text and/or in the R Markdown scripts available in the GitHub repository (https://github.com/leeoesterreich/2025_ILC_PDX_Multiomics).

### Immunohistochemistry

Formalin-fixed paraffin-embedded (FFPE) tissue from selected breast cancer PDX models was sectioned and subjected to immunohistochemical analysis of ER, PR, HER2, E-cadherin and p120. Selected models were assessed at the earliest passage/transplant generation with FFPE material available for analysis as follows: CTG-1714 (P3+1), CTG-2432 (P2+1), CTG-2518 (P2+1), CTG-2611 (P3+1), CTG-2810 (P4+1), CTG-2849 (P3), CTG-2930 (P5+1), CTG-3283 (P4), CTG-3399 (P4), CTG-3434 (P3 and P5).

The antibodies and the protocol used in this study were as follows: E-cadherin [Clone 36; Roche; dilution, ready to use (RTU); pre-treatment, CC1-S; detection, Ultraview; staining platform, Ventana Benchmark Ultra; #05905290001, RRID:AB_3714871), p120 (Clone 98; BD Biosciences; dilution, 1:200; pre-treatment, CC1-S; detection, Ultraview; staining platform, Ventana Benchmark Ultra; #610134, RRID:AB_397537), ER (Clone SP1; Roche; dilution, RTU; pre-treatment, CC1-S; detection, Ultraview; staining platform, Ventana Benchmark Ultra; #05278414001, RRID:AB_2857956), PR (Clone 1E2; Roche; dilution, RTU; pre-treatment, CC1-S; detection, Ultraview; staining platform, Ventana Benchmark Ultra; #05278392001, RRID:AB_2335976) and HER2 (Clone 4B5; Roche; dilution, RTU; pre-treatment, CC1-M; detection, Ultraview; staining platform, Ventana Benchmark Ultra; #05278368001, RRID:AB_2335975). Stained slides were interpreted by a trained breast pathologist (R.B.).

## Supplementary Material

10.1242/dmm.052710_sup1 Supplementary information

Table S5. Alteration frequency of commonly mutated breast cancer genes in basal-like NST (N=75), non-basal NST (N=40) and confirmed ILC (N=7) PDX models.
