# Supplementary material for: Identification and credentialing of patient-derived xenograft models of invasive lobular carcinoma
Source: Dis Model Mech. 2026 Apr 29;19(4):dmm052710. doi: 10.1242/dmm.052710 (PMC13225211; doi:10.1242/dmm.052710)
Supplement: Supplementary information [file dmm-19-052710-s1.pdf]

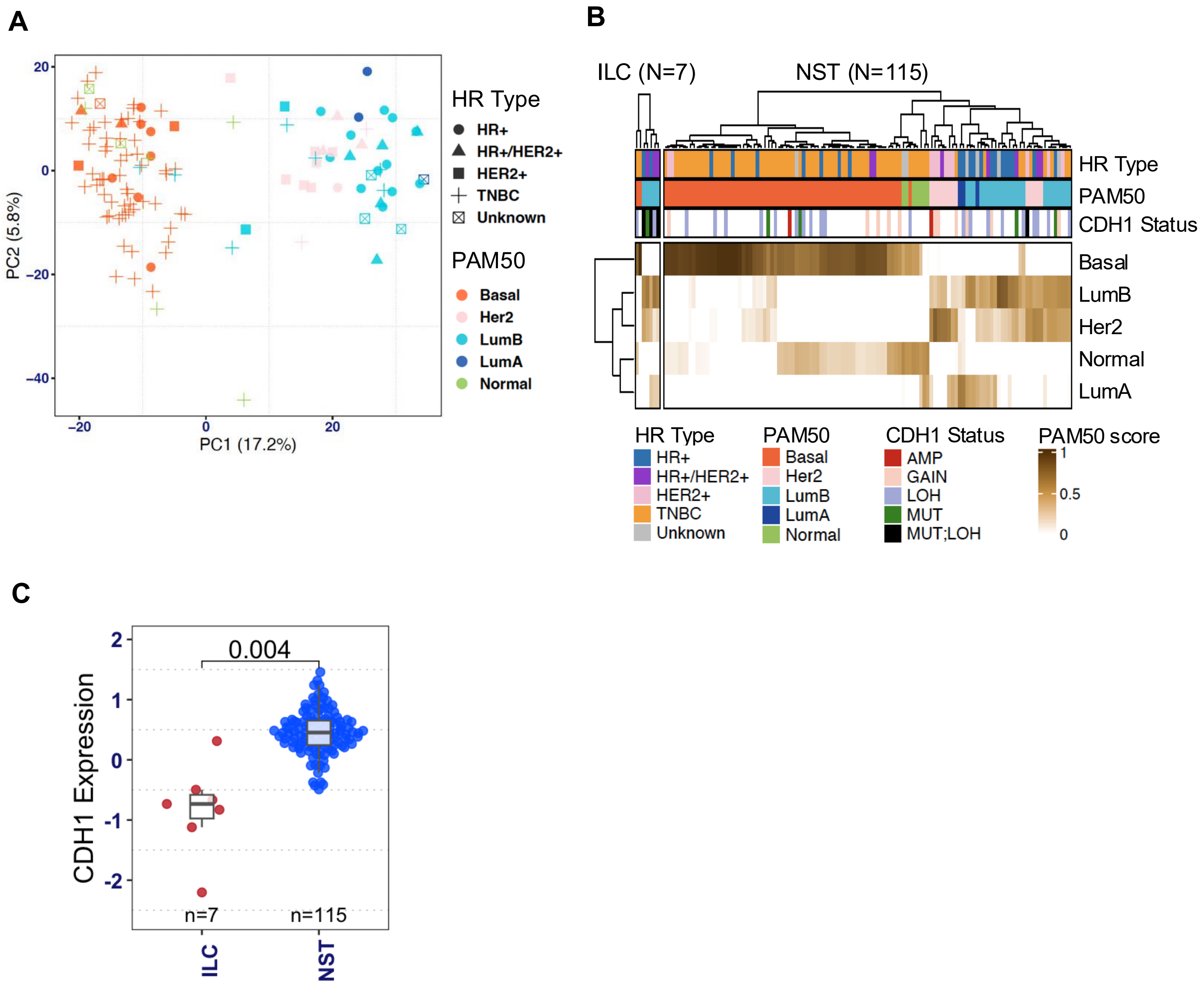

Fig. S1. Intrinsic molecular subtypes. A) HR type (hormone receptor type) indicates clinical hormone receptor and HER2 status: HR+ (ER+ and/or PR+, HER2-), HR+/HER2+ (ER+ and/or PR+, HER2+), HER2+ (ER-/PR-, HER2+), TNBC (triple-negative), or Unknown (hormone receptor status not available in clinical database). PAM50 molecular subtypes are color-coded as shown. **B)** Intrinsic molecular subtypes in putative ILC and NST PDXs. **C)** *CDH1* mRNA expression levels in confirmed ILC PDX models vs NST. Significance P-value based on T-test.

Disease Models & Mechanisms • Supplementary information

Table S1. List of PDX models (N = 13) flagged as putative ILC.

PDX Model #	Tumor status	Harvest site	Histology	Clinical Marker Status	Intrinsic Molecular Subtypes	ESR1 RNA Level	CDH1 RNA Level	CDH1 Alterations	CDH1 Mut Effect (Allele Frequency)	Selected for IHC Validation In This Study
CTG-2810	Metastatic	Pleural fluid	ILC	ER+/PR+/HER2-	LumB	high	mid	MUT;LOH	Truncating (1)	Y
CTG-3283	Metastatic	Chest	NST	ER+/PR+/HER2-	LumB	high	mid	MUT;LOH	Truncating (0.45)	Y
CTG-2432	Metastatic	Bone	NST	ER+/PR+/HER2-	LumB	high	low	MUT;LOH	Truncating (1)	Y
CTG-2611	Metastatic	Peritoneum	NST, “focally lobulated'	ER+/HER2+	LumB	high	low	LOH		Y
CTG-2930	Metastatic	Liver	NST	ER+/HER2+	LumB	high	low	MUT	Truncating (1)	Y
CTG-3302	Metastatic	Chest wall	NST	ER+/PR-/HER2-	Basal	high	low	LOH		
CTG-0869	Metastatic	Chest	NST	Triple negative	Basal	low	low	LOH		
CTG-3434	Metastatic	Abdomen fluid	ILC	ER+/PR+/HER2-	LumA	mid	low	LOH		Y
CTG-1059	Metastatic	Back	NST	ER+/PR+/HER2-	Normal	mid	low			
CTG-2849	Primary	Breast	NST	Triple negative	Basal	mid	low	LOH		Y
CTG-3399	Primary	Breast	NST	Triple negative	Basal	mid	low			Y
CTG-0888	Primary	Breast	NST	Triple negative	Basal	mid	low	GAIN		
CTG-3478	Primary	Breast	NST	Triple negative	Basal	mid	low			

List of PDX Models (N = 13) flagged as putative ILC based on truncating CDH1 mutations and/or low CDH1 mRNA expression (z-score ≤ -0.5). The 'Histology' column indicates the original clinical histopathological diagnosis of the patient tumor at diagnosis. Models CTG-2810, CTG-2611, and CTG-3434 were clinically annotated with lobular features in the original pathology reports. Other models originally diagnosed as NST (No Special Type) or ductal carcinoma were identified as having ILC molecular characteristics through our multi-omic analysis, demonstrating potential reclassification.

Disease Models & Mechanisms • Supplementary information

Table S2. PDX samples selected for IHC and histomorphic validation.

PDX Model #	Collection Site	Tumor markers (Champions)	Selection Criteria	Reported Histology (Champions)
**CTG-3434**	Abdominal fluid	ER+	Low mRNA	Lobular Carcinoma
**CTG-2432**	Bone	ER+/HER2+	Mutation	Carcinoma
**CTG-2611**	Peritoneum	ER+	Low mRNA	Ductal Carcinoma “focally lobulated'
**CTG-2810**	Pleural Fluid	ER+	Mutation	Lobular Carcinoma
**CTG-2849**	Breast	TNBC	Low mRNA	Carcinoma
**CTG-2930**	Liver	ER+/HER2+	Mutation	Ductal Carcinoma
**CTG-3283**	Chest	ER+	Mutation	Carcinoma
**CTG-3399**	Breast	TNBC	Mutation	Ductal Carcinoma
**CTG-1714**	Breast	TNBC	*NST comparison*	Carcinoma
**CTG-2518**	Breast	TNBC	*NST comparison*	Ductal Carcinoma

The 'Reported Histology (Champions)' column indicates the original clinical histopathological diagnosis from the patient tumor at diagnosis, as recorded in the Champions Oncology database. 'Carcinoma' without further specification indicates that detailed subtype classification was not provided in the original pathology report. The 'Selection Criteria' column indicates the CDH1-based molecular feature that flagged each model for validation: either presence of truncating CDH1 mutation or low CDH1 mRNA expression.

Disease Models & Mechanisms • Supplementary information

Table S3. Clinical and treatment information for ILC PDX models. Pre-biopsy treatment history indicates therapies the patient received prior to the biopsy used to establish the PDX model. 'Naive' indicates treatment-naive patients.

PDX Model #	Tumor status	Harvest site	Pre-biopsy treatment history*
CTG-0869	Metastatic	Chest	Docetaxel, Capecitabine, Bevacizumab
CTG-0888	Primary	Breast	Doxorubicin, Cyclophosphamide, Paclitaxel
CTG-1059	Metastatic	Back	Doxorubicin, Cyclophosphamide, Paclitaxel
CTG-2432	Metastatic	Bone	Tamoxifen, Letrozole, Fulvestrant, Palbociclib, Capecitabine
CTG-2611	Metastatic	Peritoneum	Tamoxifen, Everolimus
CTG-2810	Metastatic	Pleural fluid	Fulvestrant, Palbociclib, Everolimus, Exemestane
CTG-2849	Primary	Breast	Naive
CTG-2930	Metastatic	Liver	Doxorubicin, Cyclophosphamide, Paclitaxel, Tamoxifen, Paclitaxel, Fulvestrant
CTG-3283	Metastatic	Chest	Doxorubicin, Cyclophosphamide, 5-Fluorouracil, Paclitaxel, Tamoxifen, Letrozole (maintenance), Exemestane, Fulvestrant, Anastrozole, Everolimus, Experimental XPO1 inhibitor, Eribulin, Megestrol, Experimental nucleoside analogue, Palbociclib, Vinorelbine
CTG-3302	Metastatic	Chest wall	Goserelin, Doxorubicin, Cyclophosphamide, Paclitaxel, Anastrozole, Ixabepilone, Fulvestrant, Palbociclib
CTG-3399	Primary	Breast	Paclitaxel, Doxorubicin, Cyclophosphamide
CTG-3478	Primary	Breast	
CTG-3434	Metastatic	Abdomen fluid	Gemcitabine, Paclitaxel, Epirubicin, Tamoxifen, Anastrozole, Letrozole, Palbociclib, Nab-paclitaxel, Fulvestrant, Eribulin

Table S4. H&E histopathological analysis of PDX models. Histological evaluation was performed by a trained breast pathologist on FFPE sections from the indicated passages. NA = not available (no H&E slides available for analysis).

PDX Model #	Passage	Growth pattern	Any lobular-like pattern	Grade	Necrosis	Nucleoli	Plamacytoid	Apocrine
CTG-1714	P4	Solid	None	High grade	Yes	Prominent	No	No
CTG-2432	P2+1	Solid	None	High grade	Yes	Conspicuous	Yes	No
CTG-2518	P2+1	Solid	None	High grade	Yes	Prominent	No	Yes
CTG-2611	P4	Solid	Slight (some single cell growth)	High grade	Yes	Conspicuous	No	No
CTG-2810	P5	Solid	None	High grade	Yes	Prominent	No	Yes
CTG-2849	P3	NA	NA	NA	NA	NA	NA	NA
CTG-2930	P6	Solid	Slight	High grade	Yes	Prominent	No	Yes
CTG-3283	P5	Solid	None	High grade	Yes	Prominent	No	Yes
CTG-3399	P4	Solid	None	High grade	No	Prominent	No	No
CTG-3434	P4	Duct formation	None	Intermediate	Yes	Conspicuous	No	No

Table S5. Alteration frequency of commonly mutated breast cancer genes in basal-like NST (N=75), non-basal NST (N=40) and confirmed ILC (N=7) PDX models.

Available for download at
https://journals.biologists.com/dmm/article-lookup/doi/10.1242/dmm.052710#supplementary-data

Disease Models & Mechanisms • Supplementary information
